# IL-10 predicts the prognosis of patients with hepatitis B virus-related acute-on-chronic liver failure combined with spontaneous bacterial peritonitis

**DOI:** 10.3389/fmed.2023.1188300

**Published:** 2023-09-26

**Authors:** Linxiang Liu, Nanxi Xiao, Peng Chen, Xuan Zhu

**Affiliations:** ^1^Department of Gastroenterology, The First Affiliated Hospital of Nanchang University, Nanchang, Jiangxi, China; ^2^Jiangxi Clinical Research Center for Gastroenterology, Nanchang, Jiangxi, China

**Keywords:** interleukin 10, hepatitis B virus, acute-on-chronic liver failure, prognosis, COSSH-ACLF II score

## Abstract

**Background:**

Spontaneous bacterial peritonitis (SBP) is common in patients with hepatitis B virus-related acute-on-chronic liver failure (HBV-ACLF). The prognostic value of interleukin-related serum markers for patients with ACLF is coming to the fore. However, there is an unmet need to predict the survival of such patients. We aimed to analyze the independent predictors of 28- and 90-day mortality in HBV-ACLF patients with SBP.

**Methods:**

This was a retrospective study that included 368 patients with HBV-ACLF. In the SBP group, logistic regression analysis was used to understand the independent predictors of mortality at 28-day and 90-day. The accuracy of prediction was analyzed using the area under the receiver operating characteristic curve (AUROC). Finally, decision curve analysis (DCA) was used to determine the clinical utility value.

**Results:**

Interleukin 10 (IL-10) levels were statistically significantly different between the HBV-ACLF group with SBP and without. Aspartate aminotransferase (AST), serum sodium, IL-10 and vasoactive drug treatment were independent risk factors for 28-day mortality. International normalized ratio (INR), AST and IL-10 were independent risk factors for 90-day mortality. IL-10 combined with the Chinese Severe Hepatitis B Study Group-ACLF II score (COSH-ACLF IIs) had excellent performance in predicting 28- and 90-day mortality (AUCs: 0.848 and 0.823, respectively). DCA analysis suggests promising clinical utility.

**Conclusion:**

IL-10 is an independent predictor of mortality at 28- and 90-day in HBV-ACLF patients with SBP and predictive performance is improved when combined with COSH-ACLF IIs.

## Introduction

Acute-on-chronic liver failure (ACLF) is a life-threatening syndrome and is also considered a complex syndrome associated with high short-term mortality. Without timely and effective treatment, the mortality rate of ACLF can reach 50–90% (1). Worldwide, approximately 350 million people are infected with hepatitis B virus (2). ACLF caused by hepatitis B virus infection accounts for the majority of cases in Asia Pacific, especially in China (3). Therefore, there is an urgent need to find a simple, early detectable and reproducibly measurable biomarker to predict and stratify the prognosis of HBV-ACLF patients.

Systemic inflammation and susceptibility to infection are characteristic pathophysiological features of patients with ACLF (4). Immune status is an important factor affecting the prognosis of ACLF, and the potential value of interleukin-related serum markers for the prognosis of ACLF patients mentioned in some studies makes serum interleukins promising in terms of prognostic value (5, 6). Moreover, bacterial infection is one of the most common complications of ACLF, which often leads to subsequent hepatorenal syndrome (HRS), longer hospitalization practices, and ultimately high mortality in patients (7, 8). Spontaneous bacterial peritonitis (SBP) is one of the most common infections in patients with cirrhosis and also increases the risk of developing ACLF (9). However, the prognosis of HBV-associated ACLF patients with SBP is not yet known, and this is an urgent challenge to be addressed.

A variety of classical models have been used by clinicians to assess the functional status of the liver and the priority of liver transplantation. The model for end-stage liver disease (MELD), MELD-sodium (MELDNa), Child-Turcotte-Pugh (CTP) grading and COSSH-ACLF IIs, in patients with ACLF of different etiologies, the above models have reflected different prognostic values (10, 11). Recently, the hypothesis that immune dysfunction is part of the pathogenesis of ACLF patients has also been proposed (12, 13). However, it is not clear whether the prognosis of HBV-ACLF patients can be predicted by inflammatory factors or their combination with other models.

In the present study, we aimed to find a biomarker with independent predictor significance that, when combined with other models, could accurately predict 28- and 90-day mortality in patients with HBV-ACLF with SBP.

## Methods

### Study patients

This is a single-center retrospective observational study cohort that included patients admitted to the First Affiliated Hospital of Nanchang University from January 2021 to June 2022. Patients were included when they fulfilled these criteria: (1) ≥18 years old, (2) diagnosed with HBV-associated ACLF (defined by the APASL) and (3) clinical information is available. Exclusion criteria included (1) Combined with other causes of hepatitis, (2) hepatocellular carcinoma, (3) previous liver transplantation, (4) complications with other severe chronic extrahepatic diseases, and (5) infection with HIV or receiving immune-suppressive medication. Our study was approved by the Ethics Committee of the First Affiliated Hospital of Nanchang University (IIT [2021] 09).

### Definition

The diagnostic criteria for ACLF are based on the 2019 Asia-Pacific Association for the Study of the Liver (APASL) consensus (3) and are defined as follows: patients presenting with acute liver injury as evidenced by jaundice (serum bilirubin ≥5 mg/dL) and coagulation dysfunction [international normalized ratio (INR) 1.5 or prothrombin activity <40%] that is complicated within 4 weeks by clinical ascites and/or encephalopathy and previously diagnosed or undiagnosed chronic liver disease/cirrhosis (3). Briefly, the diagnosis of SBP was defined as a polymorphonuclear leukocyte (PMN) value >250 mm^3^ in the absence of any obvious infectious etiology or secondary peritonitis (14). Artificial liver therapy refers to the fact that the removal of toxins from plasma through the use of processes including adsorption and filtration will improve the clinical status of patients with liver failure. Such devices include hemodialysis, albumin dialysis and therapeutic plasma exchange. Child-Turcotte-Pugh score was computed based on albumin, ascites, serum bilirubin, HE and PT (15). The MELD formula was: 3.8 × log (bilirubin [mg/dL]) + 11.2 × log (INR) + 9.6 × log (creatinine [mg/dL]) + 6.43 (16). MELDNa formula was: (0.025 × MELD × (140–Na)) + 140 (17). COSSH-ACLF IIs = 1.649 × ln (INR) + 0.457 × HE score (HE grade: 0/1, 1–2/2 and 3–4/3) + 0.425 × ln (neutrophil [10^9^/L]) +0.396 × ln (TB [umol/L]) + 0.576 × ln (serum urea [mmol/L]) + 0.033 × age (10).

### Data collection and follow-Up

The following demographic and clinical data were collected from the patient’s electronic medical record system. Demographic variables mainly include gender, age, region, etc. Clinical data mainly include etiology, clinical laboratory tests, imaging examinations, etc. Clinical data also included inflammatory factors such as IL-1β, IL-6, IL-8, IL-10 and TNF-α. Laboratory parameters were measured in all subjects using fasting venous blood samples within the first 24 h of admission. Within 24 h of admission, a diagnostic paracentesis is performed on patients with the presence of ascites. All HBV-ACLF patients were followed up for 28 and 90 days and mortality was measured at 28, 90, and 180 days.

### Statistical analysis

Continuous variables are shown as the mean and standard deviation (SD) or median and interquartile range (IQR), while categorical variables are shown as frequencies (%). We tested whether the explanatory variable had an interaction and found no significant interactions within the included variables. Student’s *t*-tests or Mann–Whitney U test were performed for group comparisons. The diagnostic accuracy of rebleeding was assessed by receiver operating characteristic (ROC) analysis. Areas under the ROC curves (AUCs) were compared by the method of DeLong et al. All levels of significance were set at a two-sided 5% level. All analyses were performed in R 4.1.0 (R Project for Statistical Computing, Vienna, Austria). The R statistical packages gtsummary tidyverse, performance, pROC, rms and compareGroups, were used to model construction and statistical analysis. The source file “stdca.r” was obtained from the website www.mskcc.org, which was used to draw the Decision Curve Analysis (DCA) curves.

## Results

### Baseline characteristics and comparison between SBP patients and non-SBP patients

**Figure 1:**
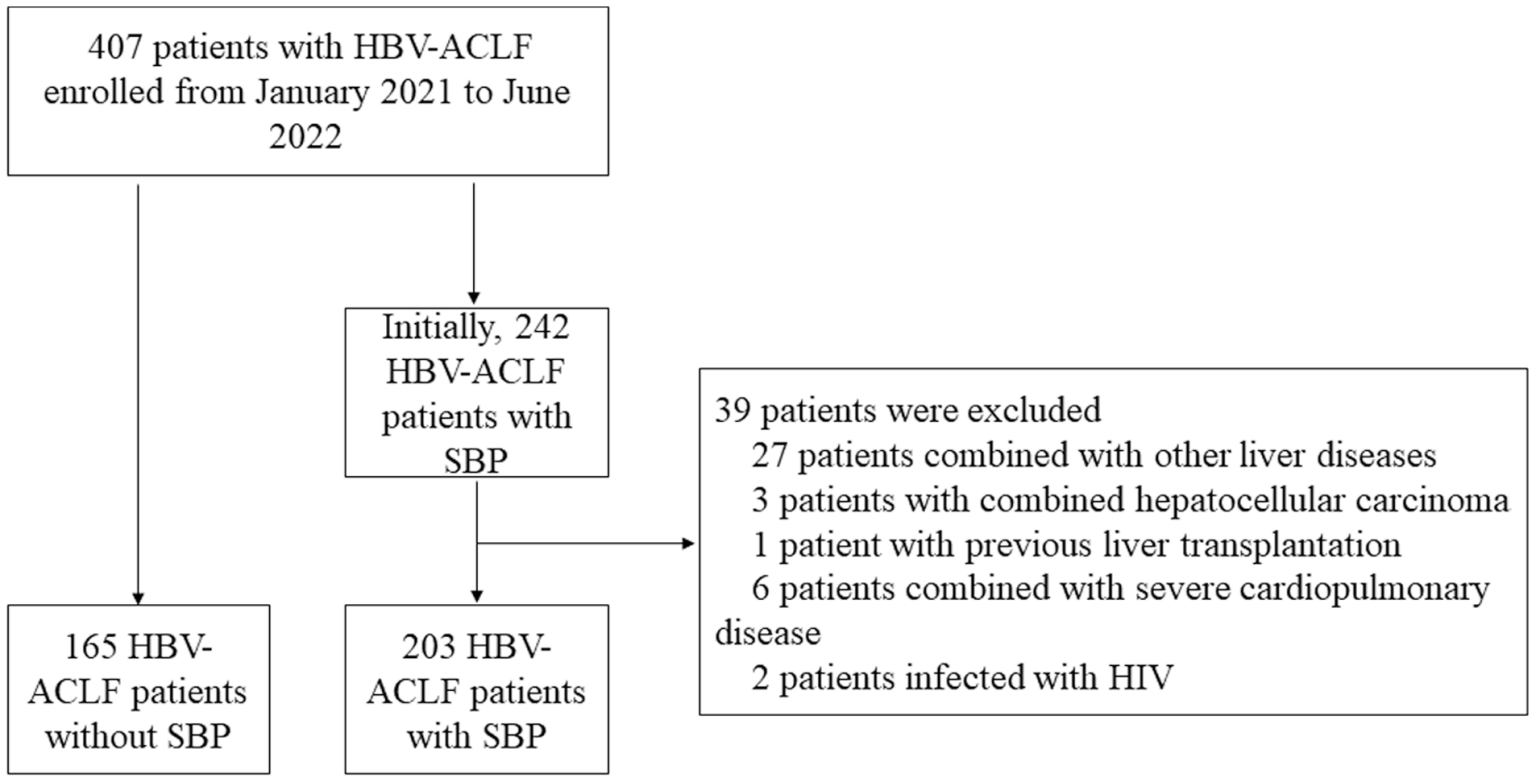
The flowchart of our study.

Figure 1 shows the flowchart of this study. As shown in Table 1, the entire cohort included 368 patients with hepatitis B-related ACLF, the majority of whom were male. During the follow-up, 122, 176, and 191 patients died within the 28th day, 90th day, and 180th day, respectively. Notably, patients with combined pneumonia accounted for 21.5% of the total cohort and 38.9% of patients with SBP. Due to the presence of ACLF in the patients included in the cohort, their laboratory tests such as liver function, renal function, routine blood, plasma inflammatory factors, and liver function scores were abnormal to varying degrees. Artificial liver therapy was performed in 68.8% of patients, and a few patients received vasoactive drugs, instrumental ventilation, and gastrointestinal endoscopy.

**Table 1:** Baseline characteristics and their comparison.

	The cohort *n* = 368	Without SBP *n* = 165	With SBP *n* = 203	*p*-value
Male	296 (80.4%)	127 (77.0%)	169 (83.3%)	0.168
Age (years)	48.8 ± 12.4	48.1 ± 13.0	49.4 ± 11.9	0.323
28-days mortality	122 (33.2%)	39 (23.6%)	83 (40.9%)	<0.001
90-days mortality	176 (47.8%)	51 (30.9%)	125 (61.6%)	<0.001
180-days mortality	191 (51.9%)	55 (33.3%)	136 (67.0%)	<0.001
Combined pneumonia	79 (21.5%)	0 (0.0%)	79 (38.9%)	<0.001
INR	2.04 (1.74–2.63)	1.93 (1.66–2.58)	2.12 (1.81–2.65)	0.01
PT (s)	22.4 (19.0–28.3)	21.2 (18.6–28.7)	22.7 (19.3–28.3)	0.15
D-dimer (mg/L)	1.99 (0.94–3.87)	1.35 (0.73–2.80)	2.66 (1.18–4.24)	<0.001
MAP (mmHg)	87.8 ± 12.8	88.2 ± 13.2	87.5 ± 12.5	0.623
White blood cell count (109/L)	7.22 ± 3.42	7.14 ± 3.62	7.29 ± 3.26	0.682
Lymphocyte count (109/L)	1.15 (0.83–1.53)	1.25 (0.86–1.53)	1.10 (0.80–1.52)	0.069
Neutrophil count (109/L)	4.30 (3.00–6.15)	4.17 (2.79–5.94)	4.50 (3.08–6.37)	0.076
Hemoglobin (g/L)	121 ± 23.6	125 ± 21.1	118 ± 25.0	0.003
Platelet count (109/L)	121 ± 59.6	136 ± 66.2	109 ± 50.7	<0.001
TBIL (μmol/L)	299 (207–380)	294 (199–382)	300 (212–376)	0.332
Albumin (g/L)	32.1 ± 4.64	32.6 ± 4.37	31.7 ± 4.82	0.07
ALT (U/L)	455 (135–1,171)	548 (154–1,268)	391 (110–913)	0.013
AST (U/L)	274 (142–696)	296 (159–791)	272 (132–647)	0.115
ALP (U/L)	159 (126–203)	156 (124–198)	162 (127–203)	0.523
GGT (U/L)	101 (68.8–143)	111 (82.0–158)	92.0 (59.0–131)	0.001
sCr (μmol/L)	64.4 (54.4–77.9)	62.7 (54.7–75.3)	65.7 (54.3–80.8)	0.175
BUN (mmol/)	3.80 (2.80–5.43)	3.70 (2.70–5.00)	4.00 (2.85–5.70)	0.066
Cholesterol (mmol/L)	2.63 (2.05–3.13)	2.77 (2.29–3.32)	2.45 (1.93–3.01)	<0.001
Triglyceride (mmol/L)	1.08 (0.72–1.50)	1.15 (0.84–1.56)	1.01 (0.64–1.42)	0.002
HDL (mmol/L)	0.23 (0.16–0.35)	0.24 (0.17–0.38)	0.21 (0.16–0.33)	0.019
LDL (mmol/L)	1.00 (0.57–1.42)	1.09 (0.63–1.50)	0.88 (0.49–1.36)	0.016
sNa (mmol/L)	140 ± 65.5	144 ± 97.7	136 ± 4.55	0.295
IL-1β (pg/mL)	10.6 (5.00–24.8)	9.49 (5.00–19.8)	11.7 (5.00–29.5)	0.031
IL-6 (pg/mL)	11.4 (6.09–24.9)	8.72 (4.80–15.6)	15.9 (7.60–40.9)	<0.001
IL-8 (pg/mL)	51.5 (28.2–93.7)	50.1 (24.2–76.2)	53.8 (31.4–103)	0.052
IL-10 (pg/mL)	3.08 (1.72–6.81)	4.06 (1.76–8.45)	2.66 (1.69–5.47)	0.031
TNF-α (pg/mL)	9.02 (2.94–17.8)	10.8 (3.59–20.2)	6.29 (2.50–14.9)	0.003
PCT (ng/mL)	0.58 (0.32–0.98)	0.49 (0.29–0.82)	0.69 (0.35–1.13)	0.002
HBeAg-positive	106 (29.0%)	44 (26.8%)	62 (30.7%)	0.487
HBV-DNA (log10 IU/mL)	4.70 (2.84–6.22)	4.67 (2.87–6.48)	4.71 (2.83–6.10)	0.921
CTP	12.0 (11.0–13.0)	12.0 (11.0–13.0)	13.0 (11.0–13.0)	<0.001
grade B		15 (9.1%)	6 (2.9%)	
grade C		150 (90.9%)	197 (97.1%)	
MELD	22.6 (19.5–26.4)	21.8 (19.0–25.4)	22.9 (19.7–27.0)	0.085
COSSH-ACLF IIs	7.32 (6.72–8.22)	7.20 (6.63–7.93)	7.49 (6.85–8.34)	0.006
MELDNa	23.2 (19.5–27.9)	22.6 (19.2–27.2)	23.6 (19.9–28.4)	0.104
Vasoactive drug treatment	28 (7.73%)	7 (4.27%)	21 (10.6%)	0.04
Device ventilation treatment	2 (0.54%)	1 (0.61%)	1 (0.49%)	1
Artificial Liver Treatment	253 (68.8%)	107 (64.8%)	146 (71.9%)	0.179
Gastrointestinal endoscopic treatment	3 (0.82%)	1 (0.61%)	2 (0.99%)	1

Data are presented as the mean ± SD, *n* (%), or median (interquartile range).

INR, international normalized ratio; PT, prothrombin time; MAP, mean artery pressure; TBIL, total bilirubin; ALT, Alanine aminotransferase; AST, aspartate aminotransferase; ALP, alkaline phosphatase; GGT, glutamyl transpeptidase; sCr, serum creatinine; BUN, blood urea nitrogen; HDL, high-density lipoprotein; LDL, low-density lipoprotein; sNa, Serum sodium; PCT, procalcitonin; HBeAg, hepatitis B virus e antigen; CTP, Child-Turcotte-Pugh; COSSH-ACLF IIs, Chinese Group on the Study of Severe Hepatitis B-ACLF II score; MELD, Model for end-stage liver disease.

In comparing the baseline characteristics of patients with and without SBP, it was found that patients with SBP had significantly higher mortality at 28, 90, and 180 days than the group without SBP. These patients with combined SBP had higher levels of INR, D-dimer, IL-1β, IL-6, TNF-α, PCT, CTP, COSSH-ACLF IIs, and more patients had used vasoactive drugs. However, their laboratory tests such as hemoglobin, platelet count, ALT, GGT, cholesterol, triglyceride, HDL, LDL, IL-10, and TNF-α levels were even lower.

### Univariate and multivariate risk factors for 28-day and 90-day mortality in HBV-ACLF patients with SBP

To avoid overfitting, correlation tests between variables-variables are performed before developing logistic regression models, and variables with correlation coefficients greater than 0.8 were excluded before developing the model (Supplementary Figure 1A). By the univariate analysis, Age, INR, PT, White blood cell count, Lymphocyte count, Neutrophil count, hemoglobin, TBIL, AST, serum creatinine, BUN, serum sodium, IL-10, HBeAg-positive, CTP, MELD, COSSHACLF IIs, vasoactive drug treatment were identified as risk factors for 28-day mortality. By multivariate logistic regression analysis, AST (OR 1.002; 95% CI 1.001–1.003; *p* < 0.001), Serum sodium (OR 0.88; 95% CI 0.79–0.98; *p* = 0.019), IL-10 (OR1.13; 95% CI 1.06–1.23; *p* < 0.001), and vasoactive drug treatment (OR 3.65; 95% CI 1.03–14.0; *p* = 0.049) were finally identified as independent risk factors for 28-day mortality (Table 2). The variance inflation factors for the variables that make up the logistic regression model of 28-day mortality were also calculated and the results showed to be within acceptable limits (Supplementary Figure 1B).

**Table 2:** Univariate and multivariate risk factors for 28-day mortality in HBV-ACLF patients with SBP.

	Univariable analysis		Multivariable analysis	
	OR(95%CI)	*p*-value	OR(95%CI)	*p*-value
Female	1.56 (0.74–3.29)	0.238		
Age	1.05 (1.02–1.08)	<0.001		
INR	4.39 (2.54–7.98)	<0.001		
PT	1.06 (1.02–1.11)	0.004		
D-dimer	1.01 (0.98–1.05)	0.565		
MAP	1.002 (0.980–1.025)	0.824		
White blood cell count	1.13 (1.04–1.25)	0.006		
Lymphocyte count	0.32 (0.17–0.56)	<0.001		
Neutrophil count	1.23 (1.11–1.38)	<0.001		
Hemoglobin	0.98 (0.97–0.99)	0.01		
Platelet count	0.996 (0.989–1.001)	0.129		
TBIL	1 0.004 (1.001–1.006)	<0.001		
Albumin	1.01 (0.96–1.08)	0.636		
ALT	1 (0.999–1.001)	0.579		
AST	1 (1.0002–1.001)	0.009	1.002 (1.001–1.003)	<0.001
ALP	0.996 (0.99–1)	0.136		
GGT	0.999 (0.996–1.001)	0.747		
sCr	1.01 (1–1.02)	0.043		
BUN	1.16 (1.05–1.29)	0.004		
Cholesterol	1.01 (0.76–1.34)	0.927		
Triglyceride	0.89 (0.61–1.27)	0.537		
HDL	2.2 (0.51–10.18)	0.292		
LDL	1.11 (0.75–1.63)	0.61		
sNa	0.94 (0.88–1)	0.037	0.88 (0.79–0.98)	0.019
IL-1β	0.998 (0.993–1.002)	0.52		
IL-6	1.002 (1.001–1.007)	0.218		
IL-8	1.001 (0.998–1.004)	0.332		
IL-10	1.09 (1.04–1.15)	0.001	1.13 (1.06–1.23)	<0.001
TNF-α	0.997 (0.982–1.001)	0.568		
PCT	1.05 (0.82–1.37)	0.667		
HBeAg-positive	0.48 (0.25–0.9)	0.024		
HBV-DNA	1.11 (0.95–1.29)	0.202		
CTP	1.34 (1.07–1.69)	0.012		
MELD	1.17 (1.1–1.26)	<0.001		
COSSH-ACLF IIs	3.93 (2.66–6.09)	<0.001		
Vasoactive drug treatment	2.67 (1.07–7.06)	0.039	3.65 (1.03–14.0)	0.049
Device ventilation treatment	1.56 (0.83–3.01)	0.173		
Artificial Liver Treatment	1.45 (0.06–37.05)	0.793		

INR, international normalized ratio; PT, prothrombin time; MAP, mean artery pressure; TBIL, total bilirubin; ALT, Alanine aminotransferase; AST, aspartate aminotransferase; ALP, alkaline phosphatase; GGT, glutamyl transpeptidase; sCr, serum creatinine; BUN, blood urea nitrogen; HDL, high-density lipoprotein; LDL, low-density lipoprotein; sNa, Serum sodium; PCT, procalcitonin; HBeAg, hepatitis B virus e antigen; CTP, Child-Turcotte-Pugh; COSSH-ACLF IIs, Chinese Group on the Study of Severe Hepatitis B-ACLF II score; MELD, Model for end-stage liver disease.

A similar statistical approach was used to identify risk factors for 90-day mortality in ACLF patients with SBP, resulting in INR (OR 24.8; 95% CI 3.57–1,134.58; *p* = 0.05), AST (OR 1.001; 95% CI 1.001–1.002; *p* = 0.01) and IL-10 (OR 1.12; 95% CI 1.06–1.25; *p* = 0.018) as independent risk factors (Supplementary Table 1), and the variance inflation factor was also calculated (Supplementary Figure 1C). Interestingly, it was both AST and IL-10 that appeared in the logistic regression models for predicting 28-day and 90-day mortality.

### The value of IL-10 in predicting mortality in HBV-associated ACLF patients with SBP

**Figure 2:**
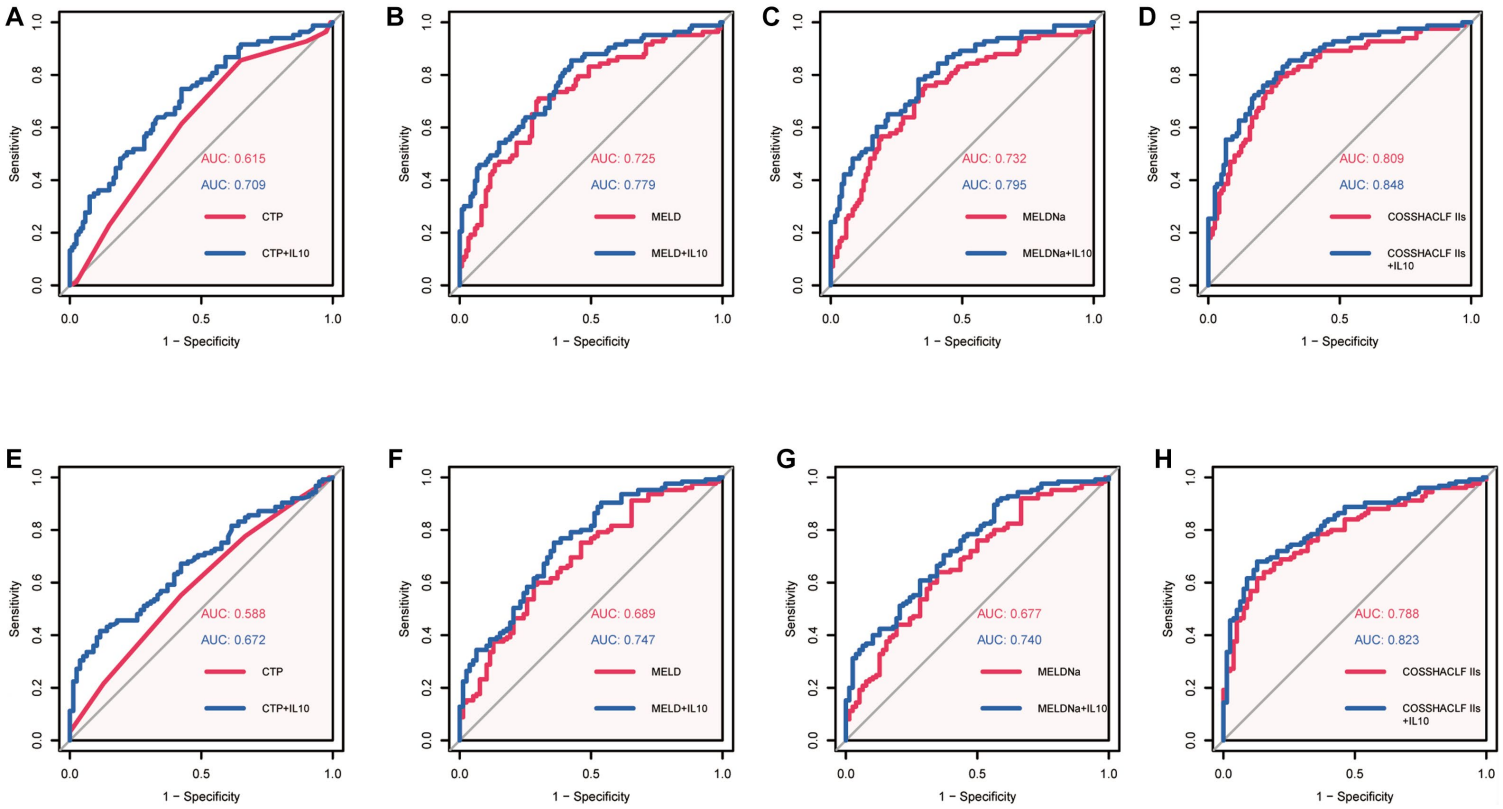
Area under the receiver operating characteristic curves (AUROC) analysis. **(A)**: AUROC of CTP predicted 28-day mortality *Vs* AUROC of CTP + IL10 predicted 28-day mortality. **(B)**: AUROC of MELD predicted 28-day mortality *Vs* AUROC of MELD+IL10 predicted 28-day mortality. **(C)**: AUROC of MELDNa predicted 28-day mortality *Vs* AUROC of MELDNa+IL10 predicted 28-day mortality. **(D)**: AUROC of COSSH-ACLF IIs predicted 28-day mortality *Vs* AUROC of COSSH-ACLF IIs + IL10 predicted 28-day mortality. **(E)**: AUROC of CTP predicted 90-day mortality *Vs* AUROC of CTP + IL10 predicted 90-day mortality. **(F)**: AUROC of MELD predicted 90-day mortality *Vs* AUROC of MELD+IL10 predicted 28-day mortality. **(G)**: AUROC of MELDNa predicted 90-day mortality *Vs* AUROC of MELDNa+IL10 predicted 90-day mortality. **(H)**: AUROC of COSSH-ACLF IIs predicted 90-day mortality *Vs* AUROC of COSSH-ACLF IIs + IL10 predicted 28-day mortality.

Our results above suggest that IL-10 may have potential prognostic predictive value. To further investigate the relationship between IL-10 and mortality in ACLF patients with SBP, we conducted an area under the receiver operating characteristic curve (AUROC) analysis to compare the prognostic value of IL-10 level and other score models. The ROC analysis gave an AUROC for the CTP, MELD, MELDNa, and COSSH-ACLF IIs scoring models in predicting mortality within 28 days and found that IL-10 combined with the other scoring models had higher predictive power than the scoring models alone (Figures 2A–D, Table 3).

**Table 3:** The comparison of predictive value between combined model and scoring model alone.

	28-day	
	AUROCs	*p*-value
IL10 + CTP *Vs* CTP	0.615 *Vs* 0.709	0.005
IL10 + MELD *Vs* MELD	0.725 *Vs* 0.779	0.036
IL10 + MELDNa *Vs* MELDNa	0.732 *Vs* 0.795	0.021
IL10 + COSSH-ACLF IIs *Vs* COSSH-ACLF IIs	0.809 *Vs* 0.848	0.016
	90-day	
	AUROCs	P-value
IL10 + CTP *Vs* CTP	0.588 *Vs* 0.672	0.012
IL10 + MELD *Vs* MELD	0.689 *Vs* 0.747	0.011
IL10 + MELDNa *Vs* MELDNa	0.677 *Vs* 0.740	0.013
IL10 + COSSH-ACLF IIs *Vs* COSSH-ACLF IIs	0.788 *Vs* 0.823	0.028

IL-10, Interleukin 10; CTP, Child-Turcotte-Pugh; COSSH-ACLF IIs, Chinese Group on the Study of Severe Hepatitis B-ACLF II score; MELD, Model for end-stage liver disease; MELDNa, MELD sodium.

Similar predictive performance was demonstrated for predicting mortality within 90 days in patients with HBV-associated ACLF with SBP (Figures 2E–H, Table 3). These results suggest that IL-10 is a reliable predictor. Furthermore, IL-10 can improve predictive performance when combined with other models. We also examined the predictive power of IL-10 and its combined prognostic score for 180-day mortality and found that it was not superior (Supplementary Figure 2).

### Clinical value analysis

**Figure 3:**
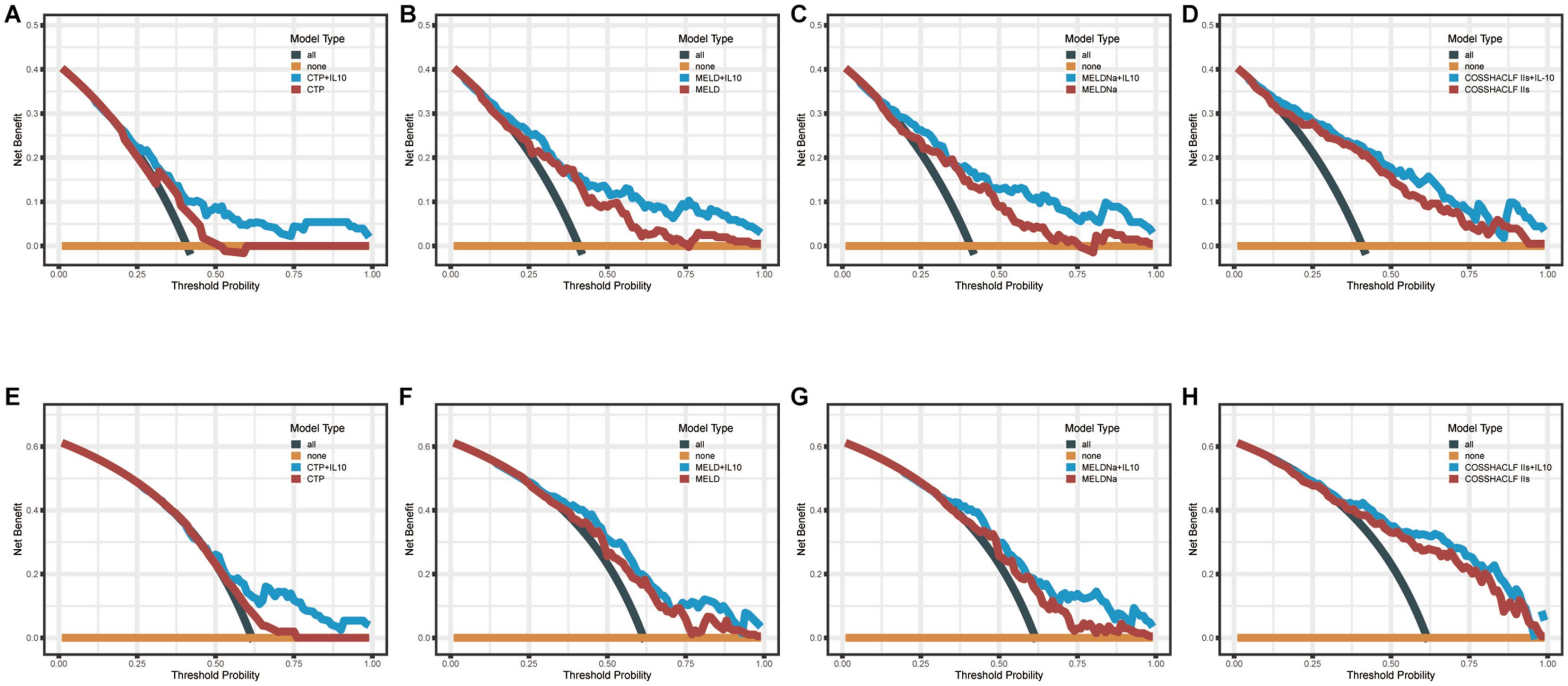
Decision Curve Analysis (DCA). **(A–D)**: DCA curves of CTP, MELD, MELDNa, COSSH-ACLF IIs and their combined IL-10 to predict 28-day mortality. **(E–H)**: DCA curves of CTP, MELD, MELDNa, COSSH-ACLF IIs, and their combined IL-10 to predict 90-day mortality.

Decision curve analysis (DCA) is a suitable method for evaluating prognostic strategies, and it offers advantages over other commonly used measurements and techniques (18). In predicting mortality in HBV-associated ACLF patients with SBP within 28 days, the net benefit of the combined IL-10 model was higher compared with the scoring model alone, indicating its high value for clinical application. Similar results were seen in the combined IL-10 models to predict mortality within 90 days (Figure 3).

## Discussion

In this study, we highlight the predictive prognostic value of IL-10 in patients with HBV-ACLF defined by APASL. Our results showed that HBV-ACLF patients with SBP had significantly higher 28-day and 90-day mortality than HBV-ACLF patients without SBP. Further, IL-10 was identified as an independent predictor of 28- and 90-day mortality in HBV-ACLF patients with SBP. Excitingly, when IL-10 was combined with MELD, MELDNa, or COSSH-ACLF IIs, it significantly improved the accuracy of predicting 28-day and 90-day mortality. Consequently, attention should be paid to this particular patient population and clinicians should come to predict the severity of the disease and optimize treatment strategies to improve patient survival.

As is generally accepted, bacterial infections are a common trigger for ACLF, and the syndrome may also increase the risk of infection (9). Specifically, SBP is also the main source of infection in this setting (9, 19). This is a complex vicious cycle, and we want to investigate the prognostic status of ACLF patients who have developed SBP. Therefore, there is an urgent need to predict the prognosis of such patients to assign individualized treatment. In our study, approximately 55.16% of patients presented with SBP. notably, it was these ACLF patients who had a diagnosis of SBP at the time of admission. Therefore, what we emphasize is the negative impact of initial bacterial inflammation on the survival of HBV-ACLF patients.

Cytokine storm is considered to be an important factor in the pathological process of ACLF (4). IL-10 is an anti-inflammatory cytokine and immunosuppressive factor that inhibits the differentiation of monocytes to antigen-presenting cells and dendritic cells and inhibits the secretion of inflammatory mediators by a variety of immune cells (20, 21). In our study, patients with HBV-associated ACLF who had SBP had higher IL-6 and lower IL-10 levels compared to those who did not have SBP. The potential mechanism could be explained by the higher level of inflammation in ACLF patients compared to those with SBP, while the expression of inflammatory factors was suppressed. Similarly, Wu et al. found that IL-6 is considered a marker of HBV-ACLF progression, also emphasizing the role of pro-inflammatory factors for ACLF progression (22). On the other hand, IL-10 was identified as an independent prognostic factor in multivariate logistic regression analysis. However, the elevation of the inflammatory cytokine IL-10 was also associated with adverse outcomes (23).

The existence of a predictive value of IL-10 rather than IL-6 for 28- and 90-day mortality in such patients is worthy of further discussion. Certain pro-inflammatory mediators, while predicting the risk of early death, may be less accurate for intermediate and overall outcomes, which are determined in part by immunoparesis (24). The degree of immunoparesis can be quantified by measuring the downregulation of the major histocompatibility molecule HLA-DR (25). Although a correlation between elevated serum IL-10 levels and the degree of HLA-DR downregulation has been observed (26).

In the present study, we found that the AUCs of COSSH-ACLF IIs score for predicting 28- and 90-day mortality were 0.809 and 0.788, respectively. When combined with IL-10, the predictive performance increased further and the difference was statistically significant. Since the COSSH-ACLF IIs was developed based on the HBV-ACLF population and contains INR, HE grade, neutrophil, TBIL, serum urea, and age, its accuracy in predicting mortality is expected in this cohort and has been validated by other cohorts as well (27).

There are some potential limitations of the study. Firstly, the results of this study were derived from a single-center retrospective study, and future multicenter prospective studies are needed to validate the findings. Second, the patients in our study were hepatitis B-related ACLF, which limits the applicability of the conclusions to alcohol-related or hepatitis C-related ACLF population. Finally, we were not able to document the dynamics of IL-10 levels. However, the purpose of our study was to highlight the impact of baseline IL-10 levels on patient prognosis.

In conclusion, IL-10 was an independent predictor of mortality at 28 and 90 days in HBV-associated ACLF patients with SBP and significantly improved the accuracy of predicting mortality when combined with other prognostic scores. The DCA analysis also suggests that IL-10 has promising clinical utility.

## Data availability statement

The original contributions presented in the study are included in the article/Supplementary material, further inquiries can be directed to the corresponding author.

## Author contributions

LL and NX contributed equally to this study. LL designed and wrote the original draft. NX analyzed the data and wrote the manuscript. PC collected the data. XZ critically revised the manuscript. All authors contributed to the article and approved the submitted version.

## Funding

This study was supported by the National Natural Science Foundation of China (grant number: 81960120).

## Conflict of interest

The authors declare that the research was conducted in the absence of any commercial or financial relationships that could be construed as a potential conflict of interest.

The reviewer DW declared a shared affiliation with the authors to the handling editor at the time of review.

## Publisher’s note

All claims expressed in this article are solely those of the authors and do not necessarily represent those of their affiliated organizations, or those of the publisher, the editors and the reviewers. Any product that may be evaluated in this article, or claim that may be made by its manufacturer, is not guaranteed or endorsed by the publisher.
