# Supplementary material for: IL-10 predicts the prognosis of patients with hepatitis B virus-related acute-on-chronic liver failure combined with spontaneous bacterial peritonitis
Source: Front Med (Lausanne). 2023 Sep 26;10:1188300. doi: 10.3389/fmed.2023.1188300 (PMC10562642; doi:10.3389/fmed.2023.1188300)
Supplement: Supplementary file 1 [file Presentation_1.ZIP › Supplementary Table 1.docx]

**Supplemental information**

Interleukin 10 predicts the prognosis of patients with hepatitis B-associated ACLF combined with SBP

**Supplementary Figure Legends:**

**Supplementary Figure 1:**

**A:** Correlation analysis among variables included in logistic regression analysis.

**B-C:** Variance inflation factors for independent risk factors associated with 28-day and 90-day mortality

**Supplementary Figure 2:**

**A-D:** ROC diagram of IL-10 and its associated score predicting 180-day mortality, where DeLong's test was also performed

Supplementary Table 1 Univariate and multivariate risk factors for 90-day mortality in HBV-ACLF patients with SBP

|  | Univariate analysis | | multivariate analysis | |
| --- | --- | --- | --- | --- |
|  | OR(95%CI) | p-value | OR(95%CI) | p-value |
| Female | 1.37 (0.64-3.1) | 0.426 |  |  |
| Age | 1.05 (1.02-1.08) | <0.001 |  |  |
| INR | 4.03 (2.24-7.71) | <0.001 | 24.8(3.57-1,134.58) | 0.05 |
| PT | 1.05 (1.01-1.1) | 0.038 |  |  |
| D-dimer | 1.001 (0.97-1.04) | 0.97 |  |  |
| MAP | 1.01 (0.99-1.03) | 0.431 |  |  |
| White blood cell count | 1.19 (1.08-1.33) | 0.001 |  |  |
| Lymphocyte count | 0.55 (0.34-0.86) | 0.012 |  |  |
| Neutrophil count | 1.31 (1.16-1.51) | <0.001 |  |  |
| Hemoglobin | 0.99 (0.98-1) | 0.103 |  |  |
| Platelet count | 0.997 (0.991-1.002) | 0.317 |  |  |
| TBIL | 1.003 (1.003-1.006) | 0.003 |  |  |
| Albumin | 1.01 (0.955-1.078) | 0.671 |  |  |
| ALT | 1.00 (0.999-1.001) | 0.343 |  |  |
| AST | 1.001 (1.001-1.002) | 0.053 | 1.001(1.001-1.002) | 0.01 |
| ALP | 1.00 (0.996-1.004) | 0.931 |  |  |
| GGT | 0.999 (0.997-1.002) | 0.843 |  |  |
| sCr | 1.01 (1.001-1.023) | 0.051 |  |  |
| BUN | 1.16 (1.04-1.32) | 0.011 |  |  |
| Cholesterol | 0.92 (0.69-1.22) | 0.576 |  |  |
| Triglyceride | 0.83 (0.58-1.18) | 0.298 |  |  |
| HDL | 1.45 (0.33-7.43) | 0.633 |  |  |
| LDL | 0.95 (0.65-1.42) | 0.809 |  |  |
| sNa | 0.94 (0.87-0.99) | 0.05 |  |  |
| IL-1β | 0.997 (0.992-1.001) | 0.231 |  |  |
| IL-6 | 1.000 (0.999-1.003) | 0.41 |  |  |
| IL-8 | 1.000 (0.997-1.002) | 0.973 |  |  |
| IL-10 | 1.09 (1.03-1.18) | 0.007 | 1.12(1.06 -1.25) | 0.018 |
| TNF-α | 1.002 (0.985-1.001) | 0.439 |  |  |
| PCT | 0.99 (0.77-1.29) | 0.914 |  |  |
| HBeAg-positive | 0.55 (0.29-1.01) | 0.054 |  |  |
| HBV-DNA | 1.13 (0.97-1.33) | 0.127 |  |  |
| CTP | 1.28 (1.03-1.59) | 0.027 |  |  |
| MELD | 1.15 (1.08-1.24) | <0.001 |  |  |
| COSSH-ACLF IIs | 3.57 (2.42-5.52) | <0.001 |  |  |
| Vasoactive drug treatment | 4.41 (1.43-19.32) | 0.021 |  |  |
| Device ventilation treatment | 1.68 (0.9-3.13) | 0.103 |  |  |
| Artificial Liver Treatment | 1.51 (0.96-2.35) | 0.072 |  |  |

Abbreviations: INR, international normalized ratio; PT, prothrombin time; MAP, mean artery pressure; TBIL, total bilirubin; ALT, Alanine aminotransferase; AST, aspartate aminotransferase; ALP, alkaline phosphatase; GGT, glutamyl transpeptidase; sCr, serum creatinine; BUN, blood urea nitrogen; HDL, high-density lipoprotein; LDL, low-density lipoprotein; sNa, Serum sodium; PCT, procalcitonin; HBeAg, hepatitis B virus e antigen; CTP, Child-Turcotte-Pugh; COSSH-ACLF IIs, Chinese Group on the Study of Severe Hepatitis B-ACLF II score; MELD, Model for end-stage liver disease.
