# Supplementary figures and images for: IL-10 predicts the prognosis of patients with hepatitis B virus-related acute-on-chronic liver failure combined with spontaneous bacterial peritonitis
Source: Front Med (Lausanne). 2023 Sep 26;10:1188300. doi: 10.3389/fmed.2023.1188300 (PMC10562642; doi:10.3389/fmed.2023.1188300)

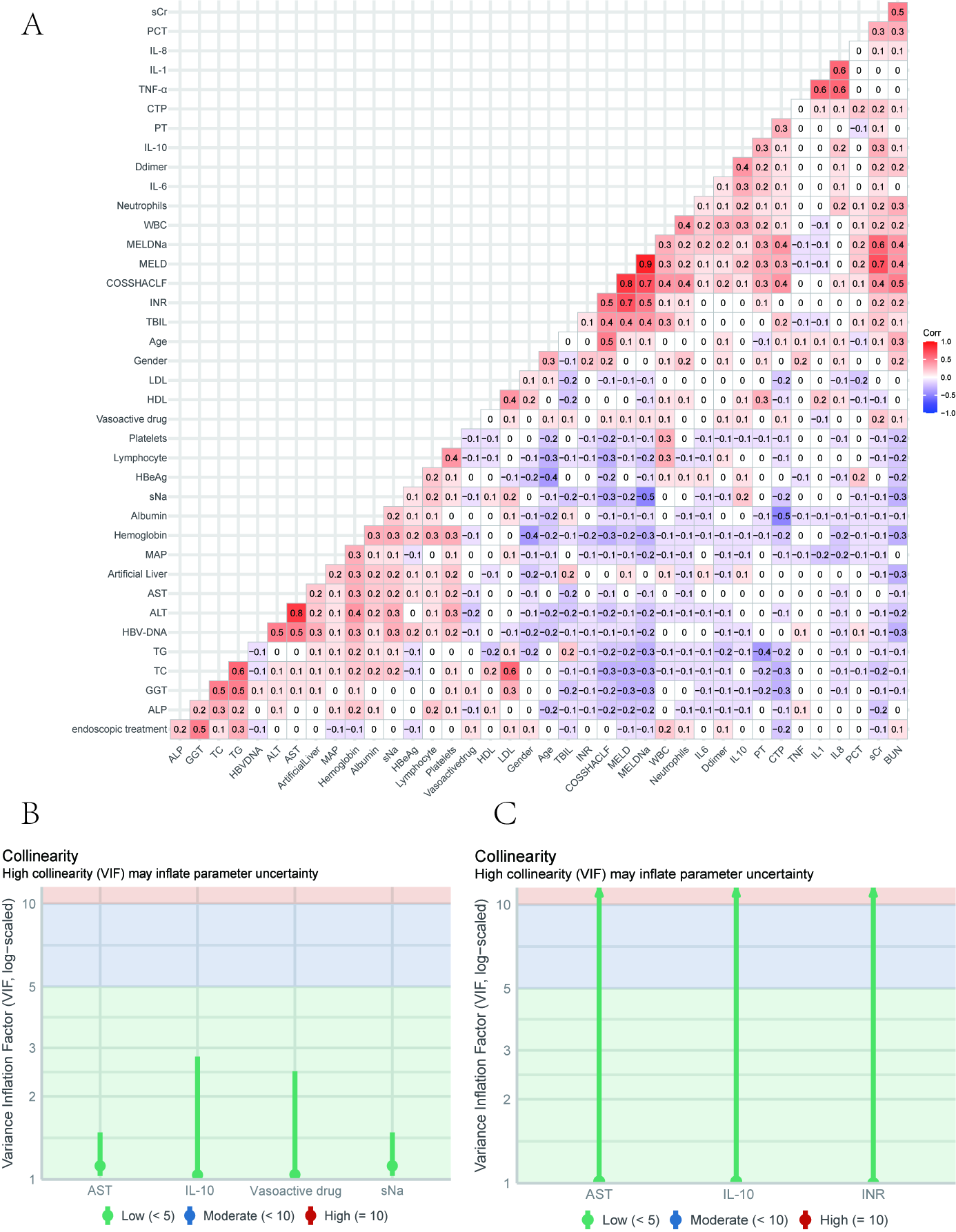

Supplement: Supplementary file 1 [file Presentation_1.ZIP › Supplementary Figure 1.tif]

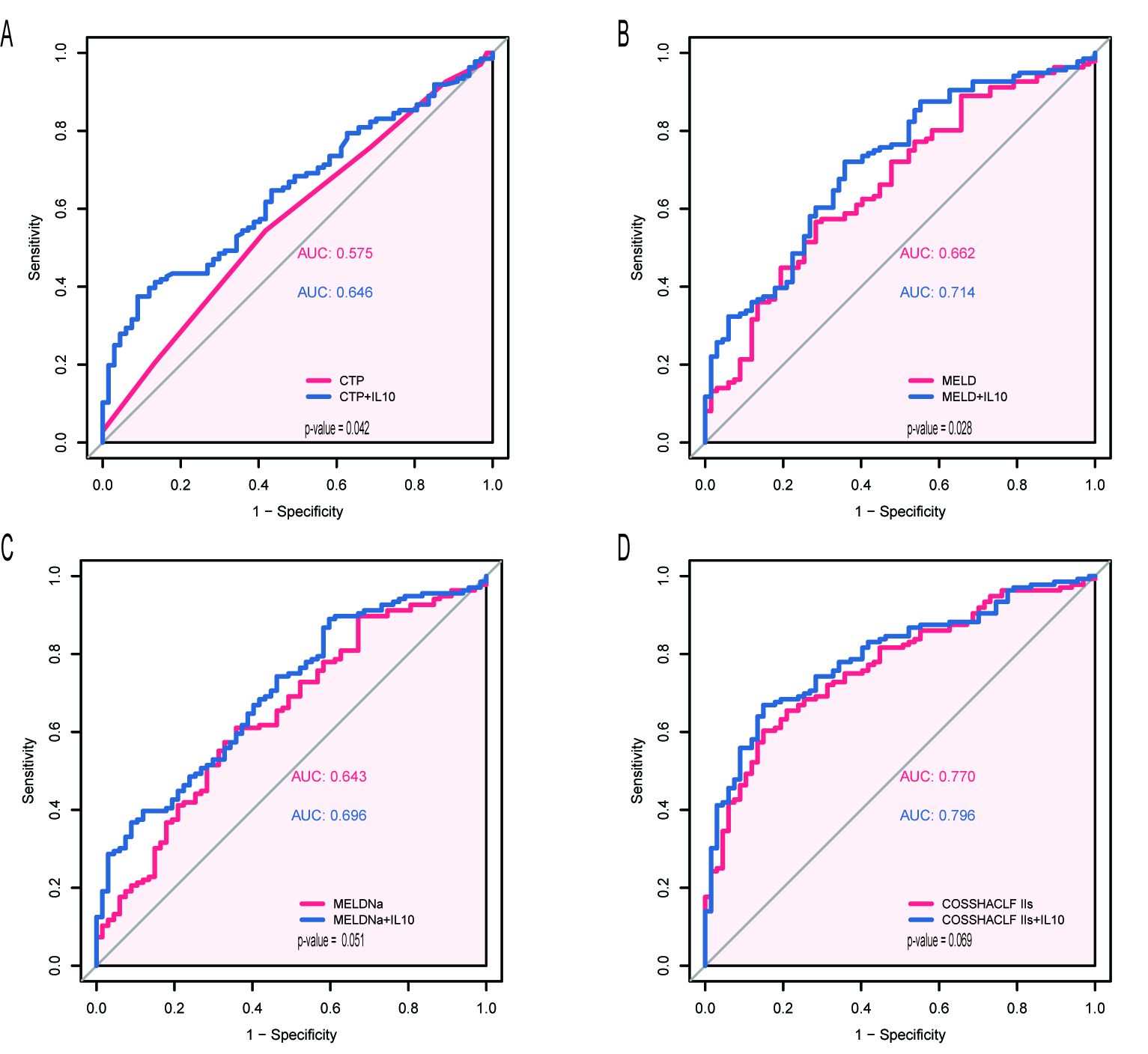

Supplement: Supplementary file 1 [file Presentation_1.ZIP › Supplementary Figure 2.tif]
